# SINGLE AND DUAL-TASK SPATIOTEMPORAL GAIT ANALYSIS OF OLDER ATHLETES PARTICIPATING IN THE NATIONAL SENIOR GAMES

**DOI:** 10.1093/geroni/igae098.4075

**Published:** 2024-12-31

**Authors:** Susan Leach, Deborah Doerfler, Jodi Schilz, Rose Vallejo, Becca Jordre, Beth Moody Jones

**Affiliations:** University of New Mexico, Albuquerque, New Mexico, United States; University of New Mexico, Albuquerque, New Mexico, United States; University of New Mexico, Albuquerque, New Mexico, United States; University of New Mexico, Albuquerque, New Mexico, United States; University of South Dakota, Vermillion, South Dakota, United States; University of New Mexico, Albuquerque, New Mexico, United States

## Abstract

One purpose of this study was to gather normative spatiotemporal gait parameters in older athletes and determine differences under three walking conditions: Forward Usual Single Task, Forward Usual Dual Task, and Forward Fast Single Task. Post hoc analyses were explored to determine a) the impact of a cognitive dual task during usual speed on gait parameters b) to determine the impact of a change in gait speed from usual to fast on gait parameters. A secondary purpose was to determine if Open, Closed or Continuous sport category influenced gait parameters. It was hypothesized that gait parameters would change with the addition of a dual task, with increased speed, and sport category. Regarding methodology, participants included 335 athletes 50 years or older in this single center cross-sectional, quasi-experimental study. Spatiotemporal gait parameters were quantified using ProtoKinetics’ Zeno Walkway system. A multivariate analysis of variance compared gait parameters under three walking conditions. Post-hoc analyses determined the impact of a cognitive dual task during usual walking speed, and a change in gait speed from usual to fast. Results indicated a significant effect of walking condition on all spatiotemporal gait parameters. From single to dual task, all gait parameters were significant with the exception of stride width coefficient of variation. From usual to fast speed, select gait parameters were significant. Sport category also demonstrated changes in select gait parameters. To conclude, normative spatiotemporal gait parameters were established for older athletes in three walking conditions and differences identified.

